# Breast Cancer Multi‐Disciplinary Team Meetings in a Resource‐Constrained System: Are Patients Receiving the Treatment Recommended?

**DOI:** 10.1002/wjs.70405

**Published:** 2026-05-07

**Authors:** Nerushka Naidoo, Lindi Martin, Wilhelmina Conradie, Magda Heunis, Charl Petrus Smit, Jenny Edge

**Affiliations:** ^1^ Division of Surgery, Department of Surgical Sciences, Faculty of Medicine and Health Sciences Stellenbosch University, and Tygerberg Hospital Cape Town South Africa; ^2^ Division of Surgery, Department of Surgical Sciences, Faculty of Medicine and Health Sciences Stellenbosch University Cape Town South Africa; ^3^ Department of Oncology, Faculty of Medicine and Health Sciences Stellenbosch University, and Tygerberg Hospital Cape Town South Africa; ^4^ Department of General Surgery, Faculty of Health Sciences University of the Witwatersrand Johannesburg South Africa

**Keywords:** breast cancer, concordance, disease stage, multidisciplinary team meetings, primary treatment

## Abstract

**Background:**

Globally, breast cancer is a sentinel health crisis, with 2.3 million new cases annually. In comparison with HICs, the stage of presentation in LMICs is higher, with a greater number of deaths occurring due to metastatic disease. A call for action with a global strategy for breast cancer is required, with an increasing role of the multidisciplinary team meeting (MDT). Its (the MDT's) use in resource‐constrained systems is not well established. This study aimed to evaluate the outcomes of MDT decisions and determine the concordance of treatment recommendations and those received by the patient. Additionally, concordance across disease stages was assessed.

**Methods:**

Retrospective data of all adult patients newly diagnosed with breast cancer and discussed at the MDT between 01 July 2021 and 30 June 2022 were collected. Patients with recurrent breast cancer, those primarily referred for palliative care, and patients with incomplete data (treatment received “unknown”) were excluded. The decision made by the MDT and the primary treatment received by the patients were assessed to determine concordance. Rates of concordance and stage of disease were calculated using contingency tables. Descriptive statistics were computed to describe continuous and categorical data.

**Results:**

A total of 556 patients were included in the analyses. Most patients (88%) received primary treatment aligned with the MDT's recommendations. The highest level of concordance was seen in those with early breast cancer who were recommended to have primary surgery (94%), and the lowest in those with metastatic disease.

**Conclusions:**

Findings demonstrate that most breast cancer patients received the treatment recommended by the MDT. Reasons for discordance could be explored in future studies. We recommend that an MDT form part of breast patient care in South Africa (SA).

## Graphical abstract

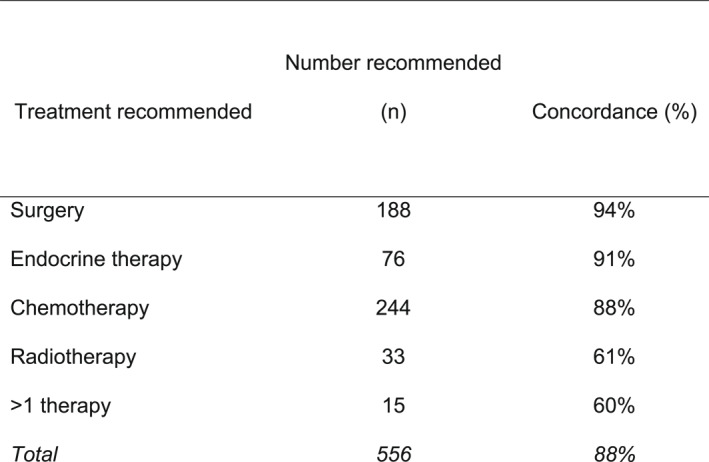

## 1 Introduction

Globally, breast cancer is a sentinel health crisis, with 2.3 million new cases annually [1]. The burden of breast cancer remains high in both low to middle‐income countries (LMICs) and high‐income countries (HICs), with inequities evident across different populations [2, 3, 4]. In the United States (US), an estimated 42,170 deaths were documented in 2025; 18.6–19.1 per 100,000, with a higher mortality rate evident in Black and Hispanic races [5].

In comparison with HICs, the stage of presentation in LMICs is higher, with a greater number of deaths occurring due to metastatic disease [6]. In South Africa (SA) at 3 years post diagnosis, the mortality in stage I–III breast cancer is 47% and in stage IV it is 80.5% [7]. Strategies to improve late‐stage presentation and subsequent mortality include population education in LMICs and screening in HICs [3].

A call for action with a global strategy for breast cancer is needed [3]. This has been supported by the World Health Organization (WHO) Global Breast Cancer Initiative (GBCI). The aim of the GBCI is to reduce breast cancer mortality by 2.5% per year, with a focus on health promotion, timely diagnoses, and comprehensive treatment [3]. Implementing resource‐stratified guidelines and integrated, multidisciplinary team meetings (MDTs) is essential to delivering equitable, high‐value care.

SA is a unique country, falling between HICs and other LMICs in terms of resources and breast cancer related mortality. Breast cancer remains the leading cancer type in females in SA, with nearly 11,000 new diagnoses in 2023 alone [8]. Although SA's mortality rate (16.1 per 100,000 [8]) outperforms much of Sub‐Saharan Africa (up to 25.9 per 100,000), a deep internal divide persists [1]. Over 80% of the SA population (62 million) relies on a public health sector stretched by limited resources, where geographical and socioeconomic barriers dictate who survives [9]. In a Sub‐Saharan study 42.4% of hospital included in the study had no access to MDTs [10].

Nietz and colleagues adapted international standards (e.g., European Society of Breast Cancer Specialists (EUSOMA) and SA National Guidelines) to define requirements for breast centers in LMICs, understanding that SA requires a tailored approach [11]. Their proposed implementation strategies included a recommended minimum patient volume for surgeons and radiologists, biannual meetings with recommendations for data recording, annual audit, and an MDT [11]. The role of the MDT is to develop a strategic plan for treatment and follow‐up, and to discuss the overall care of an individual patient [12]. The benefits of the MDT are well documented. They include increased survival, decreased variance in breast cancer patient survival, evidence‐based treatment and adherence to guidelines. MDTs also allow access to trials, improved communication between professionals and the patient, which permits the coordination of services to improve patient care, and reduce overservicing [10, 13]. That said, MDTs are only of benefit if the patient receives the recommended treatment [10].

Barriers to MDTs have been identified and categorized into three levels. System‐level barriers include infrastructure and resource limitations, such as ultrasound and pathology services [14]. Physician‐level barriers involve inadequate leadership, low motivation to participate, and excessive workload. Finally, patient‐level barriers include fragmented services and a lack of coordinated inter‐specialty care when patients present at multiple centers [15]. Additional barriers for patients adhering to diagnostic or treatment plans in SA include transport and language barriers, as well as challenges in communication and comprehension [16, 17, 18].

Tygerberg Hospital (TBH) is a tertiary referral center in Cape Town, with 1900 general beds serving over 3.6 million people [7]. The Breast Unit manages approximately 750 new patients each year [7]. Although in an LMIC, the available services are not restricted with some availability to robotic surgery, for example. TBH serves as the central hub, and patients are referred from primary and secondary health care facilities. The furthest distance a patient would have to travel to receive services at Tygerberg is 900 km.

The current screening policy allows variance due to available resources in the public and private sector; screening mammography is more prevalent in the private sector and in public screening is through breast exams. Annual screening mammograms are recommended from age 40–54; age 55 and older has a suggestion of biennial mammograms. Regular breast self‐examination and annual clinical breast examination by a health professional are advised starting from 40 [19, 20].

At the time of the study, there was no targeted therapy available for breast cancer patients treated at TBH. This was a state fund resource restriction with therapy not approved in the annual budget despite being listed as an essential treatment at the launch of the Breast Cancer Prevention and Control Policy in August 2017 [19, 20].

This study aimed to evaluate the outcomes of MDT decisions and determine the concordance of treatment recommendations and those received by the patient. Additionally, concordance across disease stages was assessed.

An MDT advocates for evidence‐based strategies ensuring improved, timely outcomes for all patients regardless of economic setting [3, 21]. This study aimed to evaluate the outcomes of MDT decisions and determine the concordance of treatment recommendations and those received by the patient. Additionally, concordance across disease stages was assessed.

## 2 Methods

This was a retrospective descriptive cohort study of newly diagnosed adult (≥ 18 years) breast cancer patients at TBH who were presented and discussed in the weekly MDT over a 1‐year period (01 July 2021 to 30 June 2022). During the MDT meetings, all present review both patient factors (comorbidities, social circumstances, risk factors, and preferences—surgery vs. chemotherapy) and cancer factors (tumor biology and stage). The MDT generally comprises 16 to 25 individuals, including pathologists, oncologists, surgeons, registrars, medical officers, medical students, and nursing practitioners. Due to understaffing, the radiology team does not participate in the MDT. A separate weekly meeting involving the breast clinic medical officers, surgeons, and radiologists is held at which, both benign and malignant cases are discussed. All breast radiological services (including stereotactic biopsies and radiology oncology) are provided at TBH with on‐site radiologists. Inevitably, there is some overlap between the meetings with some patients discussed at both. Decisions on management (i.e., treatment recommended) and treatment received by the patient are entered into an ethics board approved electronic database. The treatment recommendation is then communicated to the patient by the medical officer in the clinic.

The data assessed for this study included the primary treatment recommended at the MDT (i.e., chemotherapy, radiotherapy, surgery, or endocrine therapy) and the treatment received by the patient: systemic or surgical. Age and stage of disease were also captured.

MDT treatment decisions were compared with primary treatment received by the patient and categorized as concordant “yes”/“no.” Rates of concordance (i.e., in terms of treatment recommended and stage of disease) were calculated using contingency tables. Reasons for discordance were not explored. Descriptive statistics: median and interquartile range (IQR), where appropriate, and counts and percentages were computed to describe continuous (i.e., age) and categorical data (e.g., treatment recommended, stage of disease), respectively.

This study was approved by the Health Research Ethics Committee (HREC) of Stellenbosch University Faculty of Medicine and Health Sciences, Cape Town, South Africa (ethics reference: S22/10/197) and by TBH management. Given the retrospective nature of this study and the feasibility of contacting all patients, a waiver of consent was approved by the HREC of Stellenbosch University.

## 3 Results

Overall, 631 patients were discussed as “new” patients. Of these, 556 were included in the study. The reasons for exclusion were inadequate documentation (*n* = 47), referral for further discussion at the palliative care meeting (*n* = 24), local recurrence (*n* = 2) rather than a new primary and referral to other MDTs for nonbreast primaries (chest wall sarcoma and a patient with lymphoma) (*n* = 2). Two patients with phyllodes were included, as they were the malignant variant.

Five hundred and fifty‐six patients completed their full investigations and had complete records.

Of the 556 patients (*n* = 549 females; median age: 58, IQR: 48–67, range: 28–88 years), 488 (88%) received primary treatment concordant with that recommended in the MDT (Table 1). Most patients discussed at the MDTs were advised to receive primary chemotherapy or surgery, with fewer than 50 patients for palliative care. Most patients presented with stage 3 (35.6%), 28.9% with stage 2%, and 9.6% with stage 1. A quarter (25.9%) presented with stage 4.

**TABLE 1:** Patients receiving primary treatment as recommended by the MDT.

Treatment recommended	Number recommended (*n*)	Concordance (%)
Surgery	188	94
Endocrine therapy	76	91
Chemotherapy	244	88
Radiotherapy	33	61
> 1 therapy	15	60
Total	556	88

The greatest concordance (94%) was observed in patients whose primary treatment recommendation was surgery, followed by endocrine therapy (concordance: 91%), and chemotherapy (concordance: 88%). The lowest concordance was observed in those recommended for more than one treatment (concordance: 60%). The level of concordance was negatively associated with stage of disease, with only 77% concordance in patients with stage 4 disease (Table 2).

**TABLE 2:** Concordance by stage of disease.

Stage	Concordance (%)
1	96
2	92
3	89
4	77

## 4 Discussion

Table 3 This study presents the first SA data evaluating the concordance between MDT recommendations and actual treatment received by breast cancer patients. To our knowledge, similar data from other LMIC settings remain scarce. We demonstrated a high concordance rate of 88%, suggesting that, within the constraints of our setting, MDT recommendations are largely implemented in clinical practice.

**TABLE 3:** Concordance determined in international studies and the current study.

Study	Participant number (*n*)	Concordance
(1) Bristol, United Kingdom, 2012 [22]	210 (289 decisions)	93%
(2) Cambridge, United Kingdom, 2013 [23]	705	91.5%
(3) Melbourne, Australia, 2018 [24]	375	82%
(4) Western Australia, 2019 [25]	925	92%
(5) New South Wales, Australia [26]	835	79.4% overall
	130 breast cancer	87.3% breast cancer
(6) The Netherlands, 2022 [27]	296 in total	84.1% overall
	118 breast cancer	85.3% breast cancer
(7) Cape Town, South Africa (current study)	556	88%

The clinical significance of this finding lies in what concordance represents: alignment between evidence‐informed decision‐making and real‐world care delivery. In resource‐limited environments, attrition between planning and implementation is common [3, 28]. An 88% concordance rate, therefore, reflects not only adherence to MDT decisions but also functional coordination between surgical, oncological, radiological, and administrative services. The highest concordance (94%) was observed among patients requiring primary surgery. This likely reflects greater availability of surgical services at secondary hospitals and so fewer systemic barriers compared to patients requiring multi‐modality therapy.

Advanced‐stage patients often require multi‐modality treatment, which are more vulnerable to access limitations, delays, and system‐level constraints. Thus, the lower concordance with higher stages may represent structural inequities rather than deficiencies in MDT planning.

Consistent with other LMIC data, a large proportion of patients presented with stage III disease [28, 29]. The fact that many patients maintain clinic attendance yet still present late underscores the need for improved clinical breast pathways and streamlined referral systems [7], rather than attributing the delay solely to patient‐level factors. Health care workers should also be aware of the policy that all patients complaining of breast symptoms should be referred to the nearest regional breast unit regardless of the simplicity of the complaint [19, 20]. It is also important to acknowledge that in SA these patient‐level factors include fear of mastectomies and cultural beliefs [17, 30].

Transport limitations affect access through both cost and availability. Although a transport fund has been established at TBH to mitigate financial barriers, limited public transport infrastructure remains a structural challenge. Distance from tertiary facilities has been associated with a more advanced stage at diagnosis, particularly among older women [7, 28]. Beyond its impact on stage at presentation, geographic distance may also influence adherence to chemotherapy and radiotherapy schedules, thereby affecting long‐term outcomes.

It was mentioned earlier that at the time of the study, TBH did not have routine access to biological or targeted therapies, which are considered a mainstay of treatment in high‐income settings. In the public sector, Tamoxifen and Aromitase Inhibitors are available. Currently in the Western Cape, funding is available for only 17–20 patients per year to receive Trastuzumab (Herceptin) across both tertiary hospitals.

The poor availability of agents such as anti‐HER2 therapies or CDK4/6 inhibitors has significant implications. Internationally, these therapies have demonstrated improvements in disease‐free and overall survival in appropriately selected patients. Their inaccessibility may therefore contribute to differences in morbidity and mortality between LMIC and high‐income settings, particularly among patients with HER2‐positive or high‐risk hormone receptor‐positive disease [16, 17]. To reduce mortality, there is a persistent call for targeted therapy to be made available, with the call for funding or joint resources from the private health care sector in SA [11].

This structural limitation shapes MDT recommendations. Decisions are made within the realm of what is locally available and sustainable. In this context, variance from international guidelines should not necessarily be interpreted as diminished quality of care. Rather, it may reflect pragmatic, context‐sensitive “best possible practice” with careful allocation of resources.

The high internal concordance observed in this study suggests a cohesive and well‐functioning MDT relative to available resources. International literature emphasizes that MDT effectiveness depends not only on access to therapies but also on leadership, attendance, documentation of decisions, and implementation processes [10, 13, 21, 31]. Tools such as the MDT quality improvement framework described by Walraven et al. [15] and structured assessment instruments explored by Brown, Becker, and Young [32] provide validated methods to evaluate MDT performance beyond clinical outcomes alone. Applying such tools in our setting would allow objective benchmarking of team structure, governance, and decision quality, thereby strengthening both methodological robustness and service evaluation. Based on the internal concordance demonstrated here, the MDT would likely perform favorably in domains related to decision implementation and team cohesion.

Unpublished local data on chemotherapy adherence at TBH identified facilitators such as patient motivation and social support, whereas barriers included poor disease understanding, financial strain, and transport difficulties. These findings reinforce that improving outcomes requires both clinical excellence within MDTs and parallel strengthening of health system infrastructure.

This study has several implications for practice and policy. First, formal evaluation of MDT quality using established QI tools would provide objective validation of team performance and identify areas for improvement. Incorporating structured MDT assessment into routine governance could enhance accountability and documentation of decision implementation.

Second, expanding access to biological and targeted therapies should be prioritized at provincial and national levels. Advocacy efforts supported by local outcome data may strengthen the case for negotiated pricing frameworks. In the interim, transparent acknowledgment of therapeutic limitations in MDT discussions remains ethically important.

Third, interventions aimed at earlier detection—including community education, strengthening of primary care breast examination practices, and improved referral pathways—are likely to yield greater mortality reduction than isolated tertiary‐level improvements alone.

The current Breast Cancer Policy published in 2017 has clear guidelines, but the implementation still has room for improvement with the expected target to be reached by 2030 [19]. It is worth mentioning that the current policy for high‐risk individuals (women with a family history of breast cancer or genetic factors) should start screening 10 years before the age of the youngest relative diagnosed, or earlier, with annual mammograms [19]. NonProfit organizations provide free mammography and clinical examination to women without medical aid, particularly in underserved communities [19, 20].

Finally, future research should prospectively explore reasons for discordance between MDT recommendations and delivered care, and examine survival outcomes in this cohort. Multicenter studies would enhance generalizability and allow comparison across different resource environments within SA.

## 5 Limitations

This was a retrospective, single‐center study, which may limit generalizability. Survival outcomes were not assessed and reasons for discordance were not systematically recorded; also, data such as demographics of race, BMI, HIV status and home language were not captured. Future prospective studies incorporating survival analysis and formal MDT quality assessment would strengthen the evidence base.

## 6 Conclusions

An overall concordance of 88% was observed, consistent with rates reported in first‐world countries. The earlier the disease stage, the higher the concordance rate. We recommend that MDTs be incorporated into the standard of care for patients with breast cancer across both private and public sectors, in HIC and LMICs, as most patients ultimately receive the treatment recommended by the MDT. When subsequent changes are made to the treatment plan, we recommend re‐discussion at the MDT. Targeted therapies should be part of routine MDT consideration and they should therefore be made more available in SA.

## Author Contributions

**Nerushka Naidoo:** conceptualization, methodology, investigation, writing – original draft, writing – review and editing, project administration, resources, data curation, formal analysis. **Lindi Martin:** conceptualization, supervision, writing – review and editing, writing – original draft. **Wilhelmina Conradie:** conceptualization, writing – original draft, writing – review and editing, supervision. **Magda Heunis:** conceptualization, writing – original draft, writing – review and editing, supervision. **Charl Petrus Smit:** conceptualization, investigation, methodology, formal analysis, data curation, writing – review and editing, writing – original draft. **Jenny Edge:** conceptualization, writing – original draft, writing – review and editing, supervision.

## Funding

The project is self funded.

## Ethics Statement

This study was approved by the Health Research Ethics Committee (HREC) of Stellenbosch University Faculty of Medicine and Health Sciences, Cape Town, South Africa (ethics reference: S22/10/197) and by TBH management.

## Consent

Given the retrospective nature of this study, a waiver of consent was approved by the Health Research Ethics Committee (HREC) of Stellenbosch University.

## Conflicts of Interest

The authors declare no conflicts of interest.

## Data Availability

The data that support the findings of this study are available on request from the corresponding author. The data are not publicly available due to privacy or ethical restrictions.
